# P-1459. Racial Disparities in Treatment of Community-Acquired Pneumonia in Hospitalized Patients

**DOI:** 10.1093/ofid/ofae631.1631

**Published:** 2025-01-29

**Authors:** Ramara E Walker, Rebecca Schulte, Andrea Pallotta, Ming Wang, Victoria A Criswell, Michael Rothberg, Abhishek Deshpande

**Affiliations:** Cleveland Clinic, Cleveland, Ohio; Cleveland Clinic, Cleveland, Ohio; Cleveland Clinic, Cleveland, Ohio; Case Western Reserve University, Cleveland, Ohio; Cleveland Clinic, Cleveland, Ohio; Cleveland Clinic, Cleveland, Ohio; Cleveland Clinic, Cleveland, Ohio

## Abstract

**Background:**

Community-acquired pneumonia (CAP) is a leading cause of hospitalizations and mortality in the US. Studies have reported racial disparities in various infectious syndromes, but race is not known to be associated with antimicrobial resistance. Limited data exist on racial differences in CAP treatment in the inpatient setting. The aim of this study was to assess the impact of racial/ethnic differences on prescribing habits in hospitalized patients with CAP.

**Figure:**
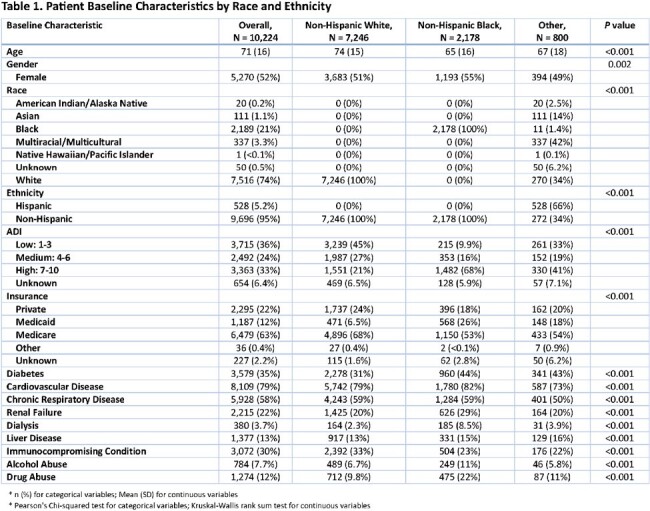

**Methods:**

This retrospective cohort study of 10,224 patients assessed associations between race/ethnicity and initiation of extended spectrum antibiotics (ESA) for non-Hispanic White (NHW), non-Hispanic Black (NHB), and other minoritized patients ≥ 18 years hospitalized at 12 Cleveland Clinic hospitals with CAP between November 1, 2022 – January 31, 2024. Parametric and non-parametric methods were used to describe demographic and clinical differences amongst the comparator groups. The association between race/ethnicity and ESA initiation was assessed using a multivariate logistic regression model adjusting for age, gender, area of deprivation index (ADI), certain comorbidities, clinical instability on day 1, and CURB65 score upon admission.

**Figure:**
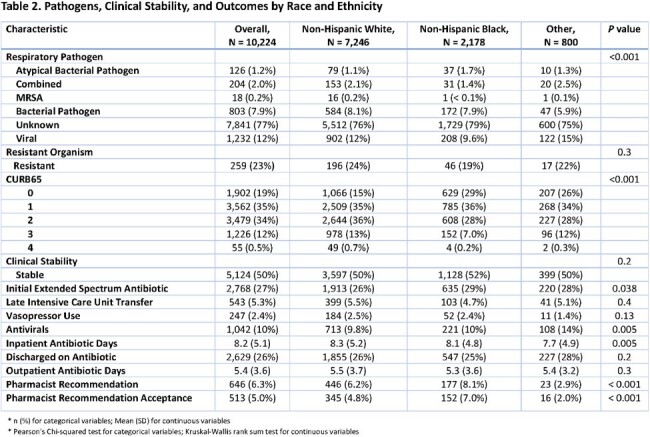

**Results:**

Compared to NHW patients, NHB patients lived in areas with higher ADI and were more likely to have Medicaid insurance, diabetes, cardiovascular disease, renal failure, and alcohol and substance abuse. Viral respiratory pathogens were more common among NHW and others versus NHB (12% vs 15% vs 10%; p < 0.001), but bacterial respiratory pathogens were similar for NHW and NHB patients with lower rates for others (8% vs 6%; p < 0.001). No racial differences were observed amongst comparator groups regarding rates of clinical stability or resistant organisms. NHB patients were initiated on ESA more often than NHW and other patients (29% vs. 26% vs. 28%, respectively; p = 0.04). In the multivariable logistic regression, NHB patients had 23% higher odds of being initiated on ESA than NHW patients (adjusted OR = 1.23; 95% CI = 1.09-1.39).

**Conclusion:**

After adjustment for clinical and sociodemographic characteristics, NHB patients with CAP were more likely to be initiated on ESA than NHW patients. Further studies are warranted to understand these differences.

**Disclosures:**

**Andrea Pallotta, PharmD**, Astra Zeneca: Advisor/Consultant|Viiv: Advisor/Consultant **Michael Rothberg, MD, MPH**, Blue Cross Blue Shield: Advisor/Consultant|CRISPER: Stocks/Bonds (Public Company)|Moderna: Stocks/Bonds (Public Company) **Abhishek Deshpande, MD, PhD**, AHRQ: Grant/Research Support|Clorox Healthcare: Grant/Research Support

